# OpenHELP (Heidelberg laparoscopy phantom): development of an open-source surgical evaluation and training tool

**DOI:** 10.1007/s00464-015-4094-0

**Published:** 2015-02-12

**Authors:** H. G. Kenngott, J. J. Wünscher, M. Wagner, A. Preukschas, A. L. Wekerle, P. Neher, S. Suwelack, S. Speidel, F. Nickel, D. Oladokun, L. Maier-Hein, R. Dillmann, H. P. Meinzer, B. P. Müller-Stich

**Affiliations:** 1 Department of General, Visceral and Transplantation Surgery, University of Heidelberg, Im Neuenheimer Feld 110, 69120 Heidelberg, Germany; 2 Division of Medical and Biological Informatics, German Cancer Research Center (DKFZ), Heidelberg, Germany; 3 Institute for Anthropomatics, Karlsruhe Institute of Technology, Karlsruhe, Germany

**Keywords:** Minimally invasive surgery, Laparoscopy, Operation phantom, Simulator, Computer-assisted surgery, Segmentation

## Abstract

**Background:**

Apart from animal testing and clinical trials, surgical research and laparoscopic training mainly rely on phantoms. The aim of this project was to design a phantom with realistic anatomy and haptic characteristics, modular design and easy reproducibility. The phantom was named open-source Heidelberg laparoscopic phantom (OpenHELP) and serves as an open-source platform.

**Methods:**

The phantom was based on an anonymized CT scan of a male patient. The anatomical structures were segmented to obtain digital three-dimensional models of the torso and the organs. The digital models were materialized via rapid prototyping. One flexible, using an elastic abdominal wall, and one rigid method, using a plastic shell, to simulate pneumoperitoneum were developed. Artificial organ production was carried out sequentially starting from raw gypsum models to silicone molds to final silicone casts. The reproduction accuracy was exemplarily evaluated for ten silicone rectum models by comparing the digital 3D surface of the original rectum with CT scan by calculating the root mean square error of surface variations. Haptic realism was also evaluated to find the most realistic silicone compositions on a visual analog scale (VAS, 0–10).

**Results:**

The rigid and durable plastic torso and soft silicone organs of the abdominal cavity were successfully produced. A simulation of pneumoperitoneum could be created successfully by both methods. The reproduction accuracy of ten silicone rectum models showed an average root mean square error of 2.26 (0–11.48) mm. Haptic realism revealed an average value on a VAS of 7.25 (5.2–9.6) for the most realistic rectum.

**Conclusion:**

The OpenHELP phantom proved to be feasible and accurate. The phantom was consecutively applied frequently in the field of computer-assisted surgery at our institutions and is accessible as an open-source project at www.open-cas.org for the academic community.

Medical innovation is connected to diligent evaluation of new medical devices with respect to applicability, relevance and safety. It relies mainly on clinical trials, animal experiments and phantom studies [1–3]. Clinical trials are clearly considered to be gold standard for clinical evaluation [4], but they are complex, cost intensive and sometimes ethically not justifiable [5, 6]. In early stages of the development of medical devices and software, studies on humans are not reasonable due to the need of further improvement prior to human application. In addition, ethical concerns and in most countries legal restrictions prohibit the use of early stage medical devices in humans [4]. Animal experiments offer realistic anatomy and surgical workflow similar to humans [7, 8]. Ethical concerns, high costs, high effort and the need for an animal testing license, which is restricted to certain proficiencies in general excluding computer scientists, mathematicians and physicists sometimes pose unsurpassable problems [9, 10]. Phantoms are easy to handle but show deficits in realism with respect to anatomy, tissue properties and motion (i.e., breathing, manipulation). This makes them only partly useful for many applications, especially when they are used for only one experiment. In addition, phantoms for single use are usually not very sophisticated. Altogether this may lead to a need for animal studies already in early stages of projects. In addition, in surgical training there is a gap between laparoscopic training devices such as box trainers and practicing on animals [11–13].

The goal of this project was to develop a reusable phantom model that combines realistic anatomy and realistic tissue properties in a cost-effective manner.

Our institutions were lacking a phantom with the specifications described above. As it was assumed that other centers have the same problem, the phantom called Open Heidelberg Laparoscopic Phantom (OpenHELP) was planned to be built as an open-source platform (available at www.open-cas.org) to share data and results in the computer-assisted surgery community.

## Materials and methods

### Development process

First, an appropriate dataset was selected. This was segmented in the second step to obtain digital models of torso, organs and structures. These digital models were refined in the third step to fit them for production. In the fourth step, the digital models of torso and organs were built using rapid prototyping techniques and in the fifth step evaluated concerning reproduction accuracy and haptic realism.

### Patient data

The OpenHELP phantom was based on an anonymized computed tomography (CT) scan of a male patient. The CT originated from a 26-year-old male patient of the University Hospital of Heidelberg hospitalized due to a car accident. The CT scan (Siemens Somatom Definition AS 40, Siemens AG, München) was performed with contrast agent in accordance with standard emergency room protocol of the university clinic of Heidelberg. The patient was chosen because no pathologies were detectable and anatomical structures were clearly distinguishable. The patients’ data were anonymized before it was used in this project.

The need for an ethics committee approval was carefully considered. Upon consultation, the local ethics committee deemed a vote in this particular case in correspondence with German legislation not necessary since the data were anonymized. Following good clinical practice, the patient was thoroughly informed regarding all the details of the planned project and written informed consent was obtained.

**Fig. 1:**
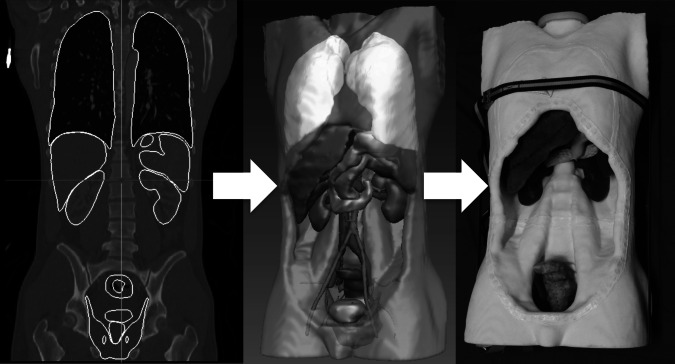
OpenHELP with visceral organs. *Left* segmented organs on the computed tomography, *middle* computer visualization of segmented organs, *right* materialized model with organs

The CT scan consisted of 351 axial slices each with a thickness of 3 mm for the whole torso excluding the limbs. Using the Digital Imaging and Communications in Medicine (DICOM) format, the CT dataset was loaded into the Medical Imaging and Interaction Toolkit (MITK, German Cancer Research Center, Heidelberg), an open-source software library that combines and extends the widespread Visualization Toolkit (VTK, Kitware Inc., New York, USA) and Insight Toolkit (ITK, Kitware Inc., New York, USA) libraries [14, 15]. The torso and the organs were segmented on each CT slice using a manual contouring tool provided by MITK. The resulting binary images were then converted into smooth 3D surfaces using the Marching Cubes algorithm of the VTK. After the segmentation process, digital surface 3D models of all organs and the torso were generated (Fig. 1).

### Production of the torso

The bones, muscles and skin were considered rigid patient structures. The torso consisted of three parts, the pelvis, the thorax including the upper abdomen and a breast shield all connected via removable plug connections to allow a modular application of the phantom. The torso was digitally cut into three elements with the help of Computer-Aided Design (CAD) methods using Creo2 (PTC Inc., Needham, USA). The muscles of the pelvic floor were segmented separately and produced out of silicone to allow a more detailed model anatomy in the lesser pelvis.

**Fig. 2:**
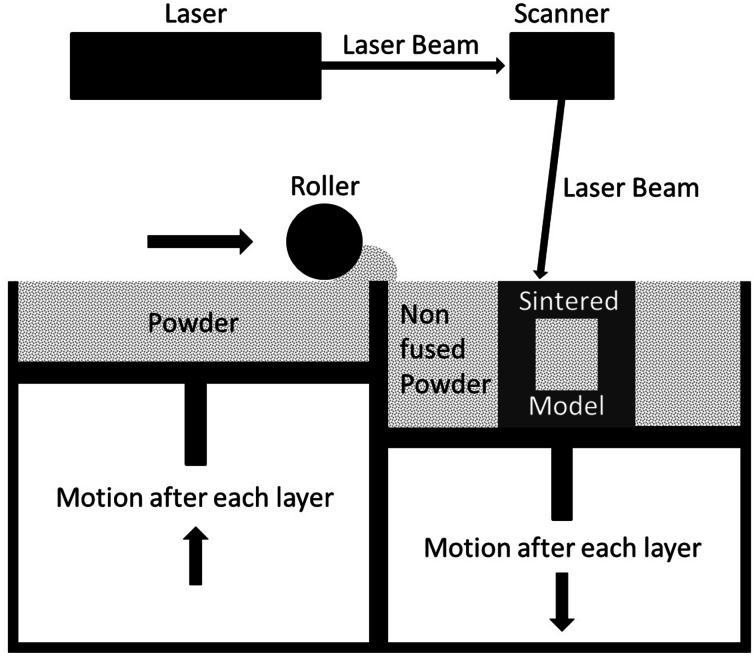
Selective laser sintering

After digital construction of the torso, several reproduction techniques were evaluated for the materialization of the torso. We created an overview of production techniques comparing their individual benefits, shortcomings and range of prices related to the requirements of the torso (Table 1) [16–20]. According to our profile of requirements, the OpenHELP torso was printed in a durable plastic (Polyamide 2200) via selective laser sintering (SLS) by an external company (3D Printwerk GbR, Fürth, Germany). SLS is an additive manufacturing layer technology using a high-power laser that fuses powder (e.g., plastic and glass) applied in layers at specific positions at the present models cross section to a solid material. The table with the model on it is lowered by the thickness of one layer for each step and a new layer of powder is applied on the rising model after a laser fuses the powder selectively. This way a rigid model rises layer by layer, surrounded by non-fused powder (Fig. 2) [17].

**Table 1:** Characteristics of rapid prototyping techniques

Profile of requirements	Selective laser sintering	Stereo lithography	Fused deposition modeling	Laminated object manufacturing
Durable	++	+	++	−
Airtight	++	++	++	++
Fluid-resistant	++	++	++	++
Light	+	+	+	+
Accurate	++	+	−	−
Smooth surface	+	++	−	−
Production speed	+	+	−	++
Maximum part size	++	++	−	+
Production cost	↑	↑↑/↑	↑↑	↓

### Production of the organs

**Fig. 3:**
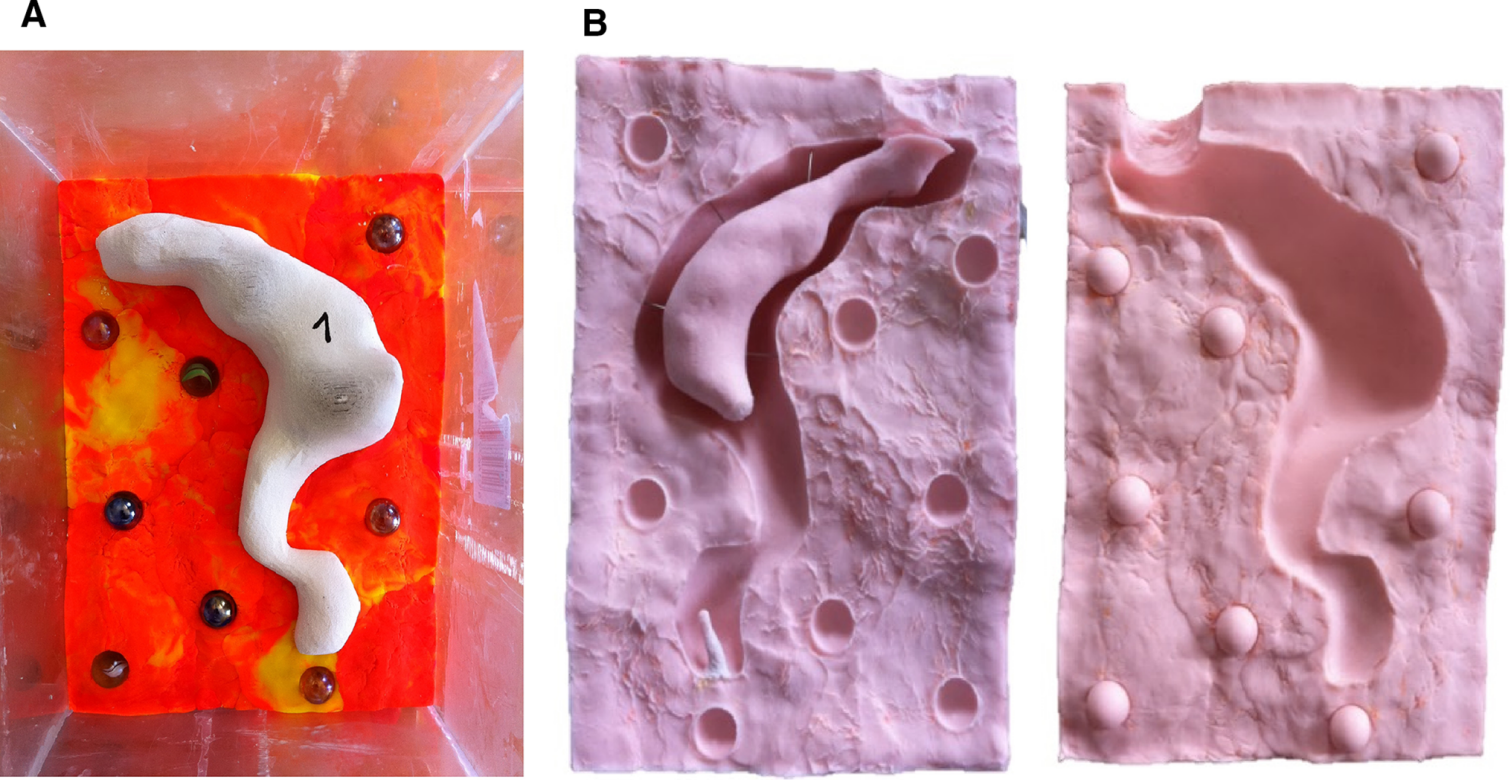
Silicone molds. **A** Gypsum rectum embedded in modeling clay with marbles, surrounded by perspex; **B** silicone mold of the rectum organ with place holder for the lumen

First, the organs were printed in gypsum using a 3D-printer (Z 450, Z Corporation, Burlington, USA). With the help of the 3D gypsum organ model, a reusable silicone mold was created (Fig. 3B). For this purpose, the gypsum organ was placed in a perspex box and molded into modeling clay until half of the models’ height was covered. The modeling clay acted as a placeholder for the second half of the mold. Spheres with a diameter of 2 cm were placed into the modeling clay until half of their diameter was covered (Fig. 3A). Fluid silicone (Mold Max 10, Smooth-On, Easton, USA) was poured over the gypsum organ and the modeling clay into the perspex box until the whole model was covered to form a negative. The cuboid consisting of the first half of the silicone mold, the gypsum model and the modeling clay were taken out of the perspex box after the silicone was cured. After removing the modeling clay and the spheres, the first half of the silicone mold and the embedded gypsum organ were placed back into the perspex box again with the silicone mold at the bottom. Then, the silicone for the second half of the mold was poured into the perspex box. After curing of the silicone, the gypsum organ was taken out of the silicone mold. The two halves of the mold could be assembled accurately with the help of the key-lock principle established by the spheres. Release spray always had to be applied before silicone was poured in order to allow easy release and opening of the mold.

Soft silicone (Smooth-On Inc., Easton, USA) was poured into the mold through a hole cut at its top. So that the surface of the organs would be smooth, a vacuum was applied in order to eliminate bubbles which entered the silicone during the preparation process. Different types and blends of silicones were used to adjust haptic characteristics and color of the artificial organs to reproduce the properties of the original organ. (Silc-Pig, Smooth-On Inc., Easton, USA). Three different types of silicones were used for the organs: Ecoflex 0010, Ecoflex 0030 and Dragon Skin FX-Pro (Table 2). Silicone additive slacker (Smooth-On Inc., Easton, USA) was used as softening agent additively. Slacker created a soft skin-like consistency depending on the amount added. Ecoflex 0010 is a very soft silicone and was used, e.g., for the bowel and the stomach. Ecoflex 0030 is slightly harder than Ecoflex 0010 and was used for the kidneys and the spleen. Dragon Skin FX-Pro was the hardest silicone among the presented three and could be used for the bladder and prostate. The Ecoflex silicones were mixable in any proportion and thus allowed an adjustment of tissue properties.

**Table 2:** Characteristics of the silicones

Characteristics	Ecoflex 0010	Ecoflex 0030	Dragon Skin FX-Pro	Mold Max 10
Type	Addition curing silicone, mix ratio of components 1A:1B			Addition curing, mix ratio 10A:1B
Viscosity (mPas)	14,000	3,000	18,000	15,000
Density (g/cm^3^)	1.04	1.07	1.06	1.15
Color	Translucent			Light pink
Pot life (min)	30	45	12	45
Demold time (h)	4	4	0.75	24
Shore hardness	00–10	00–30	A-2	A-10
Tensile strength (N/mm^2^)	0.38	1.38	2.0	3.26
Elongation at break (%)	800	900	760	375
Tear strength (N/mm)	3.92	6.78	10.88	17.83
Shrinkage (%)	<0.1	<0.1	<0.1	0.1

Except for the large and small bowel, all organs of the abdominal cavity were produced in the way as illustrated above. It was impossible to segment the bowel properly because the intestinal walls were not traceable and distinguishable in the CT scan. Therefore, another production process was developed for the bowel. A bar (diameter 2 cm) was glued onto a perspex plate using hot glue. A piece of foam insulation conduit (inner diameter approx. 3 or 4.5 cm, length 50 cm) with the same length as the rod was placed around it and was also glued on the plate. Fluid silicone was filled in the space between the bar and the foam insulation conduit. That way a lumen was generated inside the silicone tube. Four of these bowel pieces were glued together with silicone glue (Sil-Poxy, Smooth-On Inc., Easton, USA) to mimic the bowel. Different diameters were used for the large and the small bowel (outer diameter: 4.5 vs. 3 cm, respectively). Additionally a latex sheet was wrapped around the bowel and fixed with double tape representing the mesentery. The latex sheet was attached to the wall of the torso with hook-and-loop tape and therefore could be replaced easily in case of any damage.

### Simulation of the pneumoperitoneum

**Fig. 4:**
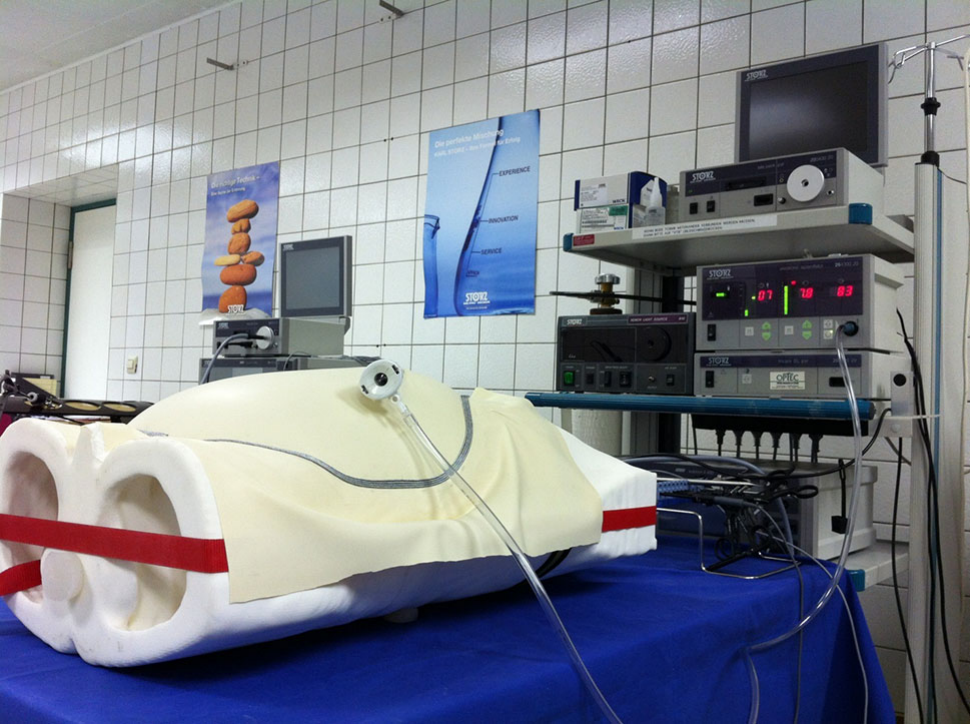
OpenHELP with established pneumoperitoneum

The phantom had the additional option to simulate pneumoperitoneum (Fig. 4). For this purpose, the torso was segmented and printed without the abdominal wall in order to replace it with an artificial inflatable skin. The artificial skin (latex sheet, thickness approx. 0.35 mm) was attached to the abdominal aperture via magnets and a metal wire. Seventy-eight neodymium magnets (diameter 10 mm, height 5 mm, adhesive force 2.4 kg/magnet) were glued into drilled holes around the aperture. Resulting surface irregularities were leveled with silicone. After covering the abdominal aperture and the surrounding magnets with a latex sheet, a metal wire was looped around the circle of magnets and closed the aperture almost airtight. Additionally, tape was attached around the abdominal aperture between the torso and the latex sheet to avoid very small leakages.

In addition to the inflatable option for the simulated pneumoperitoneum, a rigid version was developed which needed no insufflation of gas. The rigid abdominal wall was again designed with the CAD program Creo2 (PTC Inc., Needham, USA) based on the model of an insufflated abdomen. Apertures for trocars were realized in the digital 3D model, and additional trocar sites were drilled in the plastic abdominal wall later. The digital 3D abdominal wall in the shape of a dome was materialized via rapid prototyping utilizing the Fused Deposition Modeling (FDM) technique in ABS (Dimension 1200, Stratasys Ltd., Eden Prairie, USA) and resulted in a durable plastic pneumoperitoneum. In FDM, a heated ABS plastic string was placed layer by layer forming the model from the bottom up.

### Evaluation of haptic realism

**Fig. 5:**
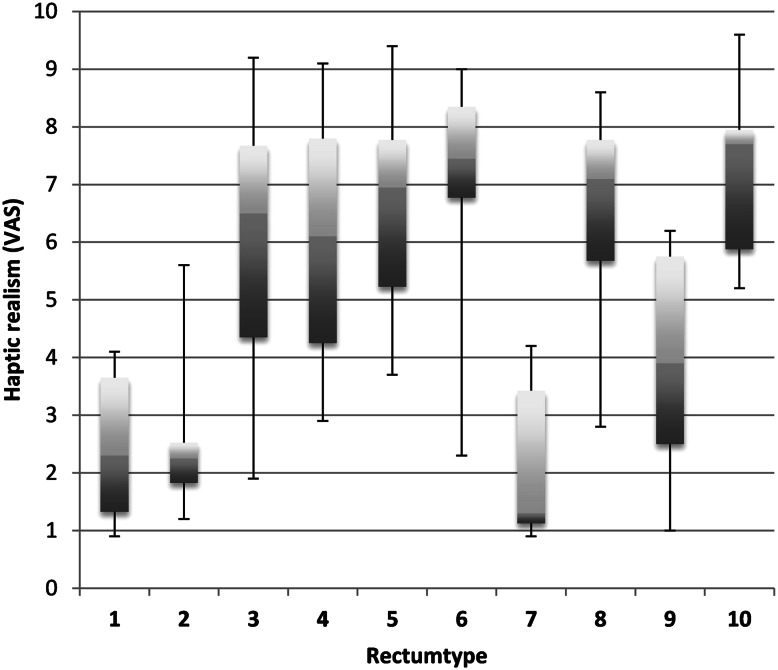
Study on haptic realism of the rectum. See Table 3 for material composition of each type; *VAS* visual analog scale

Ten generated rectum models were tested with respect to haptic realism (Fig. 5) for a current colorectal research project. The rectum was chosen due to its complex configuration. Five surgical residents and one surgical consultant were asked to evaluate ten silicone rectum models produced with different mixtures of silicone types (Table 3). The surgeons were blinded, and the rectum models were presented in a random sequence. Evaluation criteria were compressive strength and elasticity defined on a visual analog scale (VAS) ranging from zero to ten points. Zero points on the VAS were defined as “feels like a gypsum rectum,” and ten points were defined as “feels like an human rectum intraoperatively.”

**Table 3:** Ingredients of the ten silicone rectum models produced during the present study

Rectum	Silicone mixture
Rectum 1, 2	Ecoflex 0030
Rectum 3, 4, 5	Ecoflex 0010
Rectum 6	2 Ecoflex 0030:1 slacker
Rectum 7	Dragon Skin FX/Pro
Rectum 8	4 Ecoflex 0030:1 slacker
Rectum 9	5 Ecoflex 0030:1 Ecoflex 0010
Rectum 10	3 Ecoflex 0030:1 slacker

### Evaluation of reproduction accuracy

**Fig. 6:**
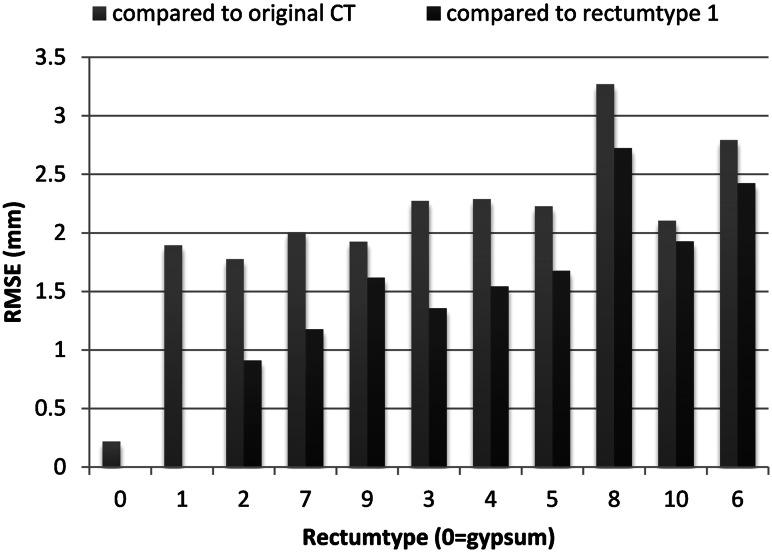
Study on reproduction accuracy of the rectum. *RMSE* root mean square error; rectum type to be compared to Table 3

The reproduction accuracy was tested exemplarily for the rectum (Fig. 6). All ten silicone rectum models (Table 3) and one gypsum rectum as reference were CT scanned with a slice thickness of 1 mm. Because of the high contrast between the rectum models and the surrounding air, the eleven CT scans of the rectum models could be segmented automatically using a basic threshold algorithm in MITK. Consequently, eleven digital surface models were obtained. The digital surface of each rectum model was registered (1) to the original rectum 3D surface of the patients’ CT and (2) to the 3D surface of the gypsum rectum via Iterative Closest Point (ICP) algorithm [21]. The root mean square error (RMSE) as a measure of reproduction accuracy describing the variance of distances and combining the average value and standard deviation was calculated by comparing the registered surfaces in MITK for each rectum.

## Results

All components of the phantom fit together seamlessly and were built in full scale.

### Torso

The torso of the phantom consisted of three parts: the pelvis, the thorax with upper abdomen and a breast shield. This enabled a modular application if the entire abdomen was not needed. The three elements were fixed together with the help of a plug connection. The dimensions of the pelvis were 252.9 × 376.5 × 206.1 mm. The dimensions of the thorax were 444.4 × 380.9 × 221.4 mm. The breast shield measured 239.0 × 245.6 × 48.8 mm. The torso had two small dorsal orifices for the passage of tubes or cables, e.g., in case organ perfusion was desired. Another two orifices were located at the neck stump and at the anus. If necessary, these orifices can be closed airtight by individually manufactured silicone plugs (Fig. 1).

### Organs

The phantom included all organs of the abdominal and thoracic cavity. The abdominal cavity of the phantom contained the liver, the spleen, the kidneys, the pancreas and the stomach. For added reality, the stomach can be produced with a lumen to make grasping with a laparoscopic instrument possible. The intestine and the mesentery were produced in the way explained above. The artificial mesentery allowed typical preparation tasks as often performed in laparoscopic surgery. The pelvis contained the rectum, the urinary bladder with prostate and seminal vesicles. Pelvic floor muscles were manufactured out of silicone to ease manipulation in the pelvis and to fix the rectum and the urinary bladder at their appropriate localization (Fig. 1).

### Simulated pneumoperitoneum

The latex sheet as a replacement for the human abdominal wall could be incised for the insertion of a standard laparoscopic port in order to inflate the artificial abdominal cavity. A maximum of 11 mmHg was achieved with a standard insufflator (Electronic Laparoflator, Karl Storz GmbH & Co. KG, Tuttlingen, Germany) at a carbon monoxide gas flow rate of 8 l/min. Thereby, a realistic dome was established and allowed laparoscopic manipulation in the abdominal cavity (Fig. 4).

### Haptic realism

The most realistic rectum in terms of haptic characteristics on the VAS (7.25; 5.2–9.6) was built with a mixture of silicone consisting of three parts Ecoflex 0030 and one part slacker as softening agent. Rectum models created out of Dragon Skin silicone or pure Ecoflex 0030 achieved poor results on the VAS (2.6; 0.9–5.6). Rectum models created out of Ecoflex 0030 with slacker as softening agent in various concentrations or pure Ecoflex 0010 generally showed the best results on the VAS (Table 3; Fig. 5). Overall haptic realism was not calculated since the goal of the study was to find the most realistic rectum.

### Evaluation of reproduction accuracy

The comparison of the surfaces of the gypsum rectum and the ten silicone rectum models with the surface of the original patients’ rectum showed an average RMSE of 0.22 mm (range of min. 3.33 × 10^−7^ and max. 3.75) for the gypsum rectum and 2.26 mm (range of min. 1.64 × 10^−5^ and max. 11.48) for the silicone rectum models. The comparison of the surfaces of the ten silicone rectum models with the surface of the gypsum rectum revealed an average RMSE of 1.62 mm (1.7 × 10^−5^–7.7 mm) (Fig. 6).

## Discussion

In this publication, we present a reusable phantom model combining realistic anatomy and realistic tissue properties. Every anatomical structure of the phantom could be correlated to the original CT scan of the patient. Therefore, OpenHELP was equal to human anatomy in terms of accurate shapes and offered a suitable environment with realistic silicone organs. Printing the model in three parts allowed individual assembly depending on the specific requirements. For example, if only the pelvic part of the phantom is needed, the thoracic part can be detached.

According to the comparison of the various rapid prototyping techniques, the torso model was printed via SLS (Table 1). This technique promised to produce durable, airtight, fluid-resistant and accurate models with smooth surfaces at low costs [16–20]. It was possible to model tissue properties. Furthermore, the production of reusable silicone molds allowed easy and cheap reproduction and replacement of worn out organs. Organs and other anatomical structures could be attached and detached based on user preference and necessity. The lacking embedment of the silicone organs in a material comparable to connective tissue left a margin for further development and would extend the application scope of the OpenHELP phantom.

The possibility for the establishment of a pneumoperitoneum was a special characteristic of the phantom. The created working space for the surgeon in the abdominal cavity was similar to the one in real surgery. However, it must be stated that the creation of the pneumoperitoneum with magnets was rather elaborate and difficult to make completely airtight. Nevertheless, the achieved pressure inside the abdominal cavity was sufficient with a maximum of 11 mmHg. It had to be taken into account that the latex sheet only had a thickness of 0.35 mm and therefore needed less pressure than a human abdominal wall (12–14 mmHg) to form an adequate dome [22, 23]. Experiments where a magnetic field needed to be established, e.g., magnetic tracking experiments, were not feasible due to the use of magnets in the construction. The rigid plastic pneumoperitoneum avoided the problems with gas leakages and allowed a quick installation and removal of the abdominal wall. However, the plastic pneumoperitoneum could not be adapted to new port positions as quickly and easily as the latex pneumoperitoneum. The rectum model achieved high results in the VAS in terms of realistic haptic characteristics as the results of the study for haptic realism of the rectum verified.

**Fig. 7:**
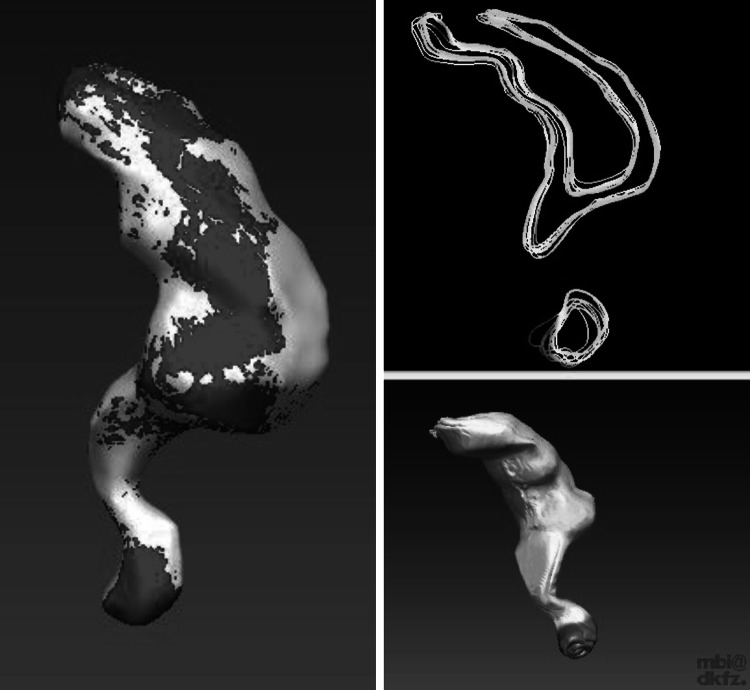
Visualization of the registered surfaces. *Green* small deviation between registered surfaces. *Red* big deviation between registered surfaces (Color figure online)

The study on the reproduction accuracy of the rectum proved that an accurate reproduction from the digital 3D model to the silicone organ was guaranteed. Differences of approximately 2 mm between the digital and the silicone model were negligible particularly with regard to the overall dimensions of the rectum of 172 × 122 × 64 mm. Moreover, the difference of 2 mm very likely occurred because of the rather flexible inferior part of the rectum. If this part was not exactly positioned on the CT table as the digital model, a deviation was noticed (Fig. 7).

OpenHELP was not the first phantom developed for the evaluation of new technical methods or surgical skills. There are other groups which concentrated on establishing new surgical phantoms, partly under their own auspices, partly in cooperation with specialized companies.

The POP Trainer (Pulsating Organ Perfusion, Optimist, Austria) developed by Szinicz et al. [24] is a commonly used device at training courses for the acquisition of laparoscopic skills. Porcine organs are placed inside the POP Trainer and can get perfused. Due to the use of real organs, diathermy can be applied. In this way, short and uncomplicated interventions such as cholecystectomies are simulated. Disadvantages are the permanent need for organs from a butcher and the unrealistic anatomical surroundings, considering that the POP Trainer has the shape of a tub.

The ELITE simulator (Endoscopic–Laparoscopic Interdisciplinary Training Entity) was developed by Feussner et al. [25, 26] in cooperation with a local company (Coburger Lehrmittelanstalt, Coburg, Germany). It consists of a human-like rigid torso and visceral organs produced on a latex basis. Retroperitoneal organs are incorporated into the torso. ELITE is especially designed for natural orifice transluminal endoscopic surgery (NOTES) and laparoscopic skills training, e.g., cholecystectomy with diathermy. However, the phantom is not available open source.

The OpenHELP project focused on providing a platform for open-source improvement and development: Thus, a holistic approach was chosen instead of developing a phantom just for one cavity or one type of application. The presented modular approach allowed a flexible adaption to new projects. In addition to the production of the phantom, it was also used as a digital model for simulation or visualization purposes.

The phantom model is planned to be further developed in an open-source setting and is thus available on the website www.open-cas.org. The torso is under revision and will allow a magnet-free fixation of the pneumoperitoneum, which will enable experiments with, i.e., electromagnetic tracking. Furthermore, the new torso will have the option to attach a diaphragm to the thoracic wall so that respiratory motion can be simulated.

All data of the OpenHELP phantom data will be uploaded and constantly updated on this webpage under creative commons license for further development and free use in research settings.

The costs for the production of one phantom amounted to 5,800€ and are subject to be decreased by further development. Particularly, the torso made up most of the costs (Table 4). Fortunately, these costs were nonrecurring costs because the plastic torso was very durable. Just the silicone organs needed replacement, when they got damaged during an experiment. A whole set of them amounted to a maximum of 200€ enabled by the easy and cheap reproduction in the silicone molds.

**Table 4:** Statement of production costs (net)

Object	Costs
Torso	4,500€
Silicone—molds	700€
Silicone—organs	200€ per set
Latex—pneumoperitoneum	20€ per 1 m^2^
Further equipment (e.g., perspex, modeling clay, magnets)	400€
Total	5.800€

In summary, OpenHELP is a reusable open-source phantom model that combines realistic anatomy and realistic tissue properties and was made available free of charge for the use of the scientific community. It might be useful for surgical research in general as well as for computer-assisted surgery and laparoscopic training.
